# Supplementary material for: Src‐dependent phosphorylation of μ‐opioid receptor at Tyr336 modulates opiate withdrawal
Source: EMBO Mol Med. 2017 Aug 18;9(11):1521–36. doi: 10.15252/emmm.201607324 (PMC5666313; doi:10.15252/emmm.201607324)
Supplement: Supplementary file 1 — Appendix [file EMMM-9-1521-s001.pdf]

APPENDIX

Table of Contents

1. Appendix Supplementary Materials and Methods (Quantitative RT-PCR) .... Pages 1-2
2. Appendix Figure S1 with legend .... Pages 3-4
3. Appendix Figure S2 with legend .... Pages 5-6
4. Appendix Figure S3 with legend .... Pages 6-7
5. Appendix Figure S4 with legend .... Pages 7-8
6. Appendix Table S1 (Exact *P*-values for Figure 6).. Page 9
7. Appendix Table S2 (Exact *P*-values for Figure 7A and C) .... Page 9
8. Appendix Table S3 (Exact *P*-values for Figure EV2) .... Page 10
9. Appendix Table S4 (Exact *P*-values for Figure EV3) .... Page 10
10. Appendix Table S5 (Exact *P*-values for Figure EV4) .... Page 11
11. Appendix Table S6 (Exact *P*-values for Figure EV5) .... Page 11
12. Appendix Table S7 (Exact *P*-values for Appendix Fig S3) .... Page 12

Quantitative RT-PCR (RT-qPCR)

RNA from the LC was isolated using TRI Reagent according to the manufacturer's protocol (Molecular Research Center, Cincinnati, OH, USA). RT-qPCR experiments were performed using QIAGEN's Quantitect SYBR Green RT-PCR kit and the Bio-Rad iCycler (Bio-Rad, Hercules, CA, USA). RT-qPCR was performed according to the same procedure described by (Song *et al*, 2007). The specific primer sequences for MOR1 were: forward, 5'-CATGGCCCTCTATTCTATCGTGT-3' and reverse, 5'-CAGCGTGCTAGTGGCTAAGG-3' (Invitrogen, Carlsbad, CA, USA). The primers for the housekeeping gene glyceraldehyde-3-phosphate dehydrogenase (GAPDH) were: forward, 5'-GGTGAAGGTCGGTGTGAACG-3' and reverse, 5'-CTCGCTCCTGGAAGATGGTG-3' (Invitrogen, Carlsbad, CA, USA). A negative control without cDNA template (called water) was run simultaneously with every assay. To translate Ct values into absolute copy numbers, standard curves were obtained for MOR1 and GAPDH. Constructing the standard curve required a 10-fold serial dilution of templates. The 10-fold

serial dilution of the templates was then amplified using the same primers for RT-qPCR MOR1 and GAPDH mRNA abundance analysis. Using the standard curve, Ct values for MOR1 in each sample was translated into absolute copy numbers, and the copy number was then normalized to the absolute quantity of GAPDH from the same sample. For each mRNA sample extracted from the LC, the RT-qPCR experiment was repeated at least four times.

Appendix Figure S1

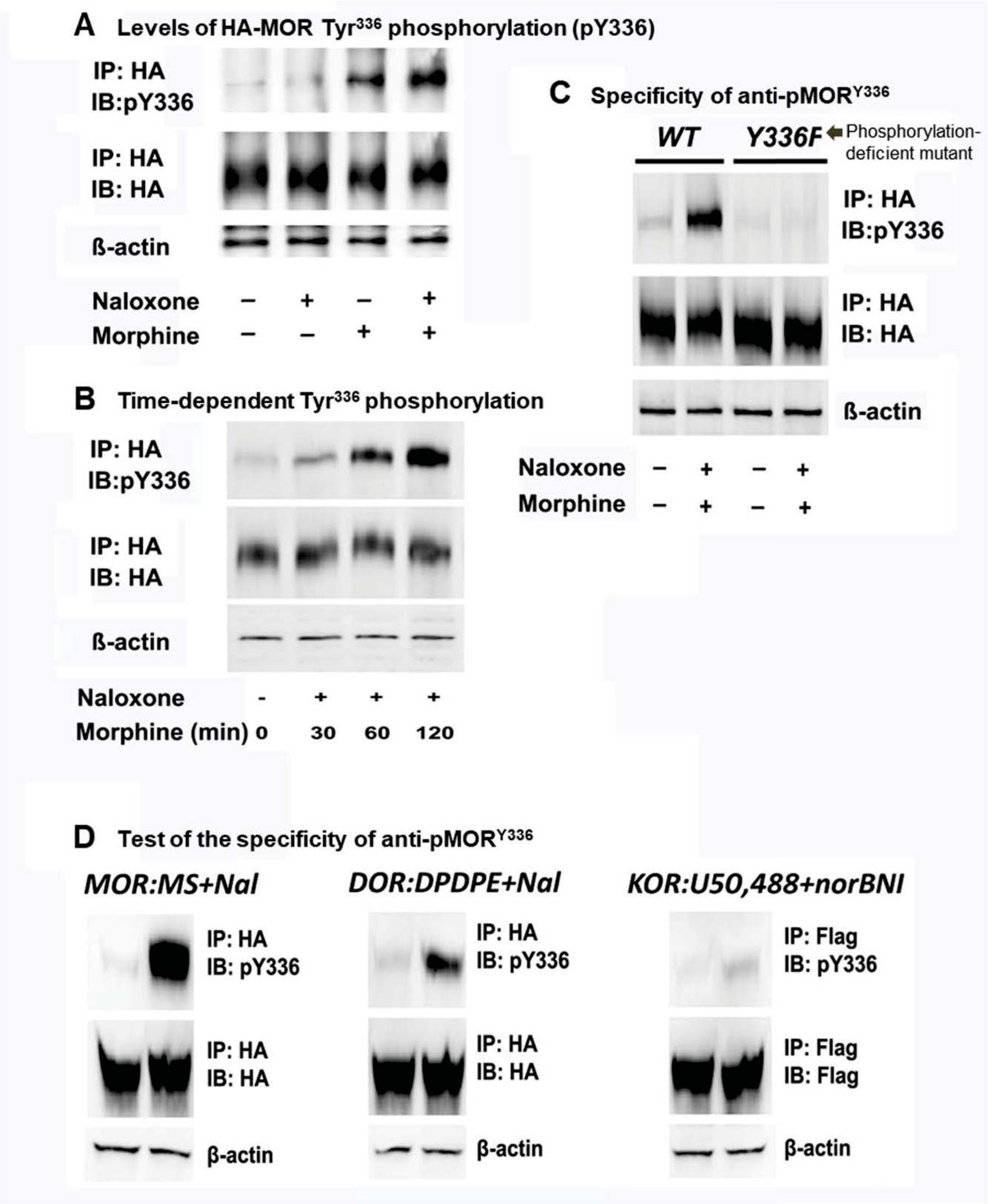

Appendix Figure S1. Increase in opioid receptor phosphorylation in the presence of antagonist after prolonged agonist treatment.

A HEK293 cells stably expressing HA-tagged wild-type (WT) MOR (WT HA-MOR) were treated with (+) or without (-) 1 μM morphine for 2 h, followed by treatment with (+) or without (-) 10 μM naloxone for 15 min. HA-MOR was immunoprecipitated and the levels of Tyr^{336} phosphorylation and total HA-tagged receptors were examined by western blot analysis as described in the Materials and Methods.

B Time-dependent analysis of the induction of Tyr^{336} phosphorylation in HEK293 cells following treatment with 1 μM morphine for the indicated periods and treatment with 10 μM naloxone for 15 min.

C Tyr^{336} phosphorylation was compared in HEK293 cells stably expressing either the WT HA-MOR or the mutant HA-MORY336F after the treatment with 1 μM morphine for 2 h, and 10 μM naloxone for 15 min.

D HEK293 cells stably expressing either HA-MOR, HA-DOR or Flag-KOR were treated with 1 μM of their agonists morphine, DPDPE or U50,488, respectively, for 2 h, and then treated with 10 μM antagonists (naloxone for MOR and DOR or nor-BNI for KOR) for 15 min. The levels of Tyr^{336} phosphorylation and the receptors were detected by IP and western blot analysis as described the in Materials and Methods. The results were normalized to the internal control β-actin, which was detected using a mouse anti-β-actin antibody (Santa Cruz sc-47778, 1:1000).

IP = Immunoprecipitate; HA = Hemagglutinin epitope; IB = Immunoblot; pY336 = Phosphorylated MOR at Tyr^{336}; WT = Wild-type MOR; Y336F = Phosphorylation-deficient MOR at Tyr^{336} (Mutation of Tyr^{336} to Phe); MOR = Mu-opioid receptor; MS = Morphine Sulfate; Nal = Naloxone; DOR = Delta-opioid receptor; DPDPE = [D-Pen2,D-Pen5]Enkephalin; KOR = Kappa-opioid receptor; norNBI = norbinaltorphimine.

Appendix Figure S2

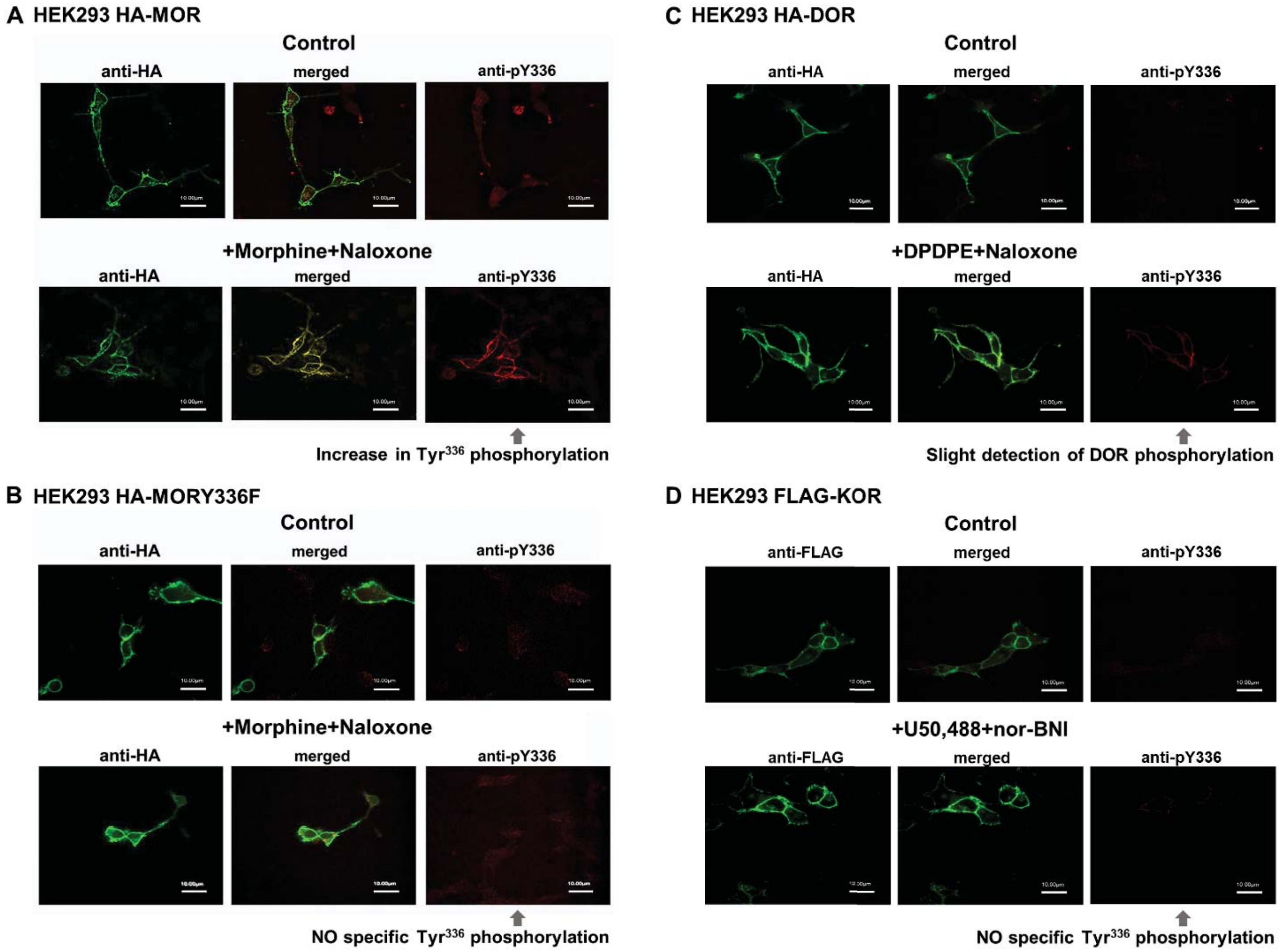

Appendix Figure S2. Immunofluorescence analysis showing the ability of an antagonist to induce an increase in Tyr^{336} phosphorylation after prolonged agonist treatment in HEK293 cells expressing various opioid receptors.

A HEK293 cells stably expressing WT HA-MOR were treated without (control) or with the selective agonist morphine (1 μM) for 2 h, followed by the treatment with naloxone (10 μM) for 15 min.

B HEK293 cells stably expressing mutant HA-MORY336F were treated without (control) or with the selective agonist morphine (1 μM) for 2 h, followed by the treatment with naloxone (10 μM) for 15 min.

C HEK293 cells stably expressing HA-DOR were treated without (control) or with the selective agonist DPDPE (1 μM) for 2 h, followed by the treatment with naloxone (10 μM).

D HEK293 cells stably expressing Flag-KOR were treated without (control) or with the selective agonist U50,488 (1 μM) for 2 h, followed by the treatment with nor-BNI (10 μM) for 15 min.

The addition of naloxone after prolonged morphine treatment is meant to mimic the *in vivo* naloxone-precipitated withdrawal. The levels of total receptor and Tyr^{336} phosphorylation were examined by immunofluorescence using anti-HA, anti-Flag or anti-$pMOR^{Y336}$ (2 μg/mL) antibodies as described in the Materials and Methods. Scale bars: 10 μm.

Appendix Figure S3

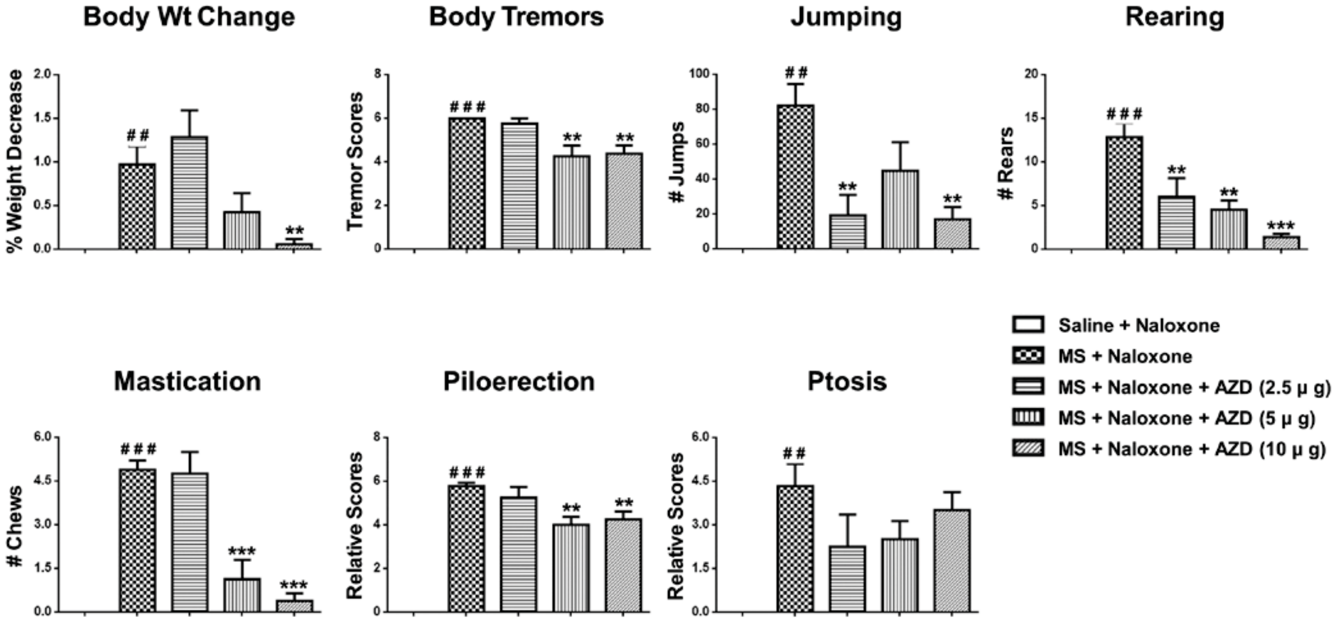

Appendix Figure S3. Effect of Src inhibition on naloxone-precipitated somatic withdrawal signs following the stereotaxic injection of different doses of AZD0530 (2.5, 5, 10 μg) into the LC of WT mice. One week after the stereotaxic injection of AZD0530 into the LC, the mice were treated with saline or progressive doses of morphine (day 1: 10 mg/kg; day 2: 20 mg/kg; day 3: 40 mg/kg; day 4: 80 mg/kg; day 5: 100 mg/kg; and day 6: 100 mg/kg). From day 1 to day 5, the animals were injected with either morphine or saline twice a day (9:00 am, 5:00 pm). On day 6, 3.5 h after the last injection of either morphine or saline on the morning, the mice were injected with different doses of AZD0530 into the LC (2.5

μg/mouse: 1.25 μg/side; 5 μg/mouse: 2.5 μg/side; 10 μg/mouse: 5 μg/side). After 30 min, the animals were injected with naloxone (10 mg/kg, i.p.), and their withdrawal signs were recorded for 30 min. The 5 μg dose of AZD0530 was the lowest that caused a consistent and significant inhibition of most of the measured naloxone-precipitated withdrawal signs (weight loss, body tremors, jumping, rearing, mastication, and piloerection). Values represent means ± SEMs. N = 9/group. Significant differences among the groups were determined using one-way ANOVAs, followed by Duncan's *post hoc* comparison. $^{***}P < 0.001$, $^{**}P < 0.01$ and $^{*}P < 0.05$ significant differences vs MS + Naloxone group. $^{\#\#\#}P < 0.001$, $^{\#\#}P < 0.01$ and $^{\#}P < 0.05$ significant differences vs Saline + Naloxone group.

Exact *P*-values are in Appendix Supplementary Table 7.

Appendix Figure S4

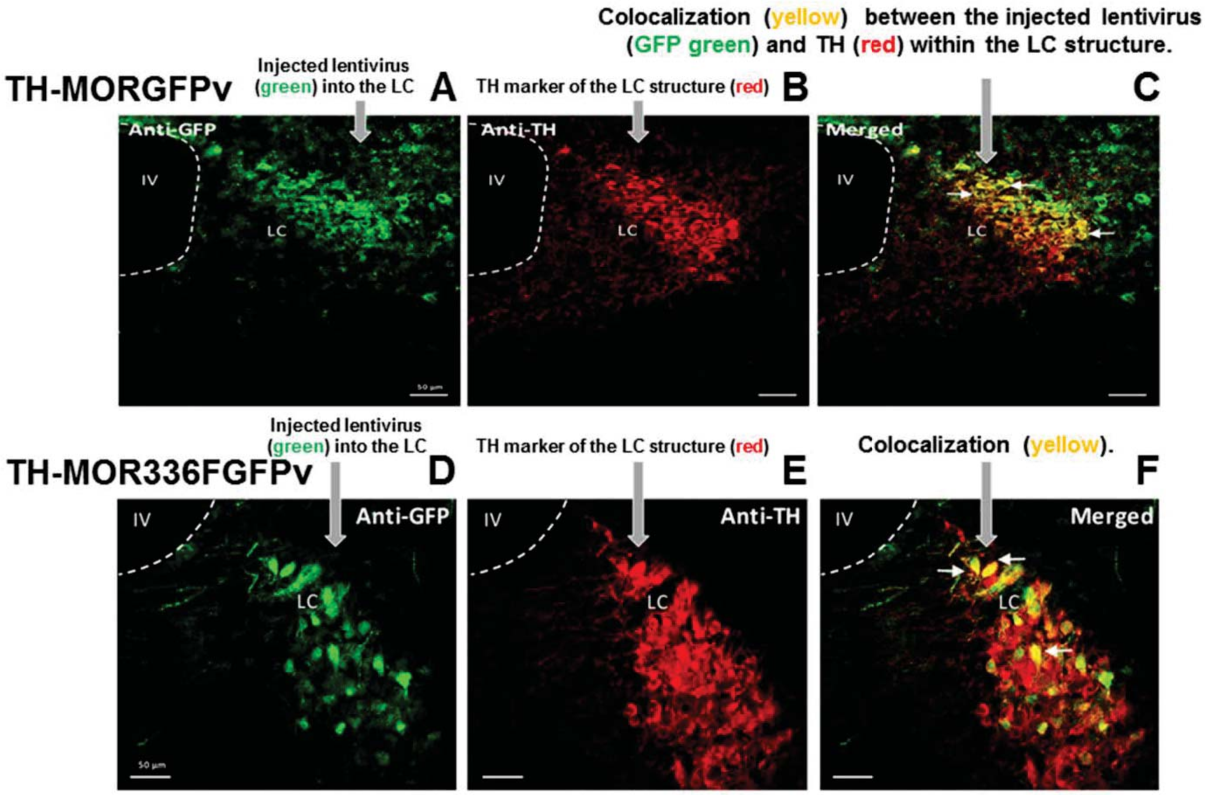

Appendix Figure S4. Localization of the TH promoter-controlled MORGFP lentivirus (TH-MORGFPv) (A-C) or MORY336FGFP lentivirus (TH-MORY336FGFPv) (D-F) expression in the LC of MOR$^{-/-}$ mice.

A Representative fluorescent micrograph of a LC transverse section from a TH-MORGFPv-expressing mouse labeled with a monoclonal GFP antibody and Alexa Fluor 488-conjugated goat anti-mouse IgG.

B Photomicrograph of the same mouse and section in (A) labeled with TH antiserum and Alexa Fluor 647-conjugated goat anti-rabbit IgG.

C Merge of the two images in (A and B) showing the colocalization of TH-MORGFPv and TH in neurons in the LC (arrows).

D Representative fluorescent micrograph of a LC transverse section from a TH-MORY336FGFPv-expressing mouse labeled with a monoclonal GFP antibody and Alexa Fluor 488-conjugated goat anti-mouse IgG.

E Photomicrograph of the same mouse and section in (D) labeled with TH antiserum and Alexa Fluor 647-conjugated goat anti-rabbit IgG.

F Merge of the two images in (D and E) showing the colocalization (yellow) of TH-MORY336FGFPv and TH in neurons in the LC (arrows). Scale bars: 50 μm.

IV = 4th ventricle; LC = Locus Coeruleus.

Appendix Table S1.

Exact *P*-values for Figure 6. Statistical differences were analyzed using ANOVAs followed by Duncan's *post hoc* comparisons.

	Figure 6 – Exact *P*-values					
	WT-Placebo vs WT-MS	$MOR^{-/-}$-GFPv-MS vs WT-MS	$MOR^{-/-}$-MORv-MS vs WT-MS	$MOR^{-/-}$-MORY336Fv-MS vs WT-MS	$MOR^{-/-}$-GFPv-MS vs $MOR^{-/-}$-MORv-MS	$MOR^{-/-}$-MORv-MS vs $MOR^{-/-}$-MORY336Fv-MS
**Body Wt Change**	* 0.0371	* 0.0335	** 0.0012	*** 0.0008	ns 0.8780	ns 0.3632
**Diarrhea**	** 0.001690	** 0.002168	*** 0.000321	** 0.001690	ns 0.214024	ns 1.00000
**Grooming**	ns 0.096919	* 0.047491	*** 0.006826	* 0.021980	ns 0.052145	ns 0.838035
**Jumping**	** 0.001103	** 0.001230	** 0.001564	** 0.001120	# 0.042529	$ 0.036738
**Locomotor Activity**	ns 0.570050	ns 0.380887	ns 0.925569	ns 0.222537	ns 0.098562	ns 0.050416
**Wet Dog Shakes**	*** 0.000645	*** 0.000523	ns 0.393488	* 0.016420	# # # 0.000832	$ $ 0.005544

vs = versus
ns = no significant differences

Appendix Table S2.

Exact *P*-values for Figure 7A. Statistical differences were analyzed using ANOVAs followed by Duncan's *post hoc* comparisons.

	Figure 7A – Exact *P*-values						
	WT-Placebo vs WT-MS	$MOR^{-/-}$-GFPv-MS vs WT-MS	$MOR^{-/-}$-MORv-MS vs WT-MS	$MOR^{-/-}$-MORY336Fv-MS vs WT-MS	$MOR^{-/-}$-GFPv-MS vs $MOR^{-/-}$-MORv-MS	$MOR^{-/-}$-GFPv-MS vs $MOR^{-/-}$-MORY336Fv-MS	$MOR^{-/-}$-MORv-MS vs $MOR^{-/-}$-MORY336Fv-MS
**MOR mRNA level (MOR/GAPDH)**	ns	*** 0.000003	*** 0.000011	*** 0.000009	# # # 0.001001	††† 0.000979	ns 0.870660

vs = versus
ns = no significant differences

Exact *P*-values for Figure 7C. Significant and strong correlation showed by Pearson's correlation coefficient r.

	Figure 7C – Exact *P*-values
	MOR vs MORY336F
**Wet Dog Shakes**	* 0.0448

vs = versus

Appendix Table S3.

Exact *P*-values for Figure EV2. Statistical differences were analyzed using ANOVAs followed by Duncan's *post hoc* comparisons.

	Figure EV2 – Exact *P*-values		
	Placebo + Naloxone vs MS + Naloxone	Placebo + Naloxone vs MS + Naloxone + AZD	MS + Naloxone vs MS + Naloxone + AZD
Body Wt Change	# 0.033251	$ 0.026498	ns 1.000000
Diarrhea	# # # 0.000120	ns 0.188503	** 0.001170
Grooming	ns 0.145950	$$ 0.007016	ns 0.123581
Jumping	# # # 0.000677	ns 0.282878	** 0.006141
Locomotor Activity	ns 0.258726	ns 0.261786	ns 0.941497
Mastication	# # # 0.000838	ns 0.487938	** 0.003151
Paw Tremors	# # # 0.000075	ns 0.344582	*** 0.000184
Wet Dog Shakes	# # # 0.000074	ns 0.267077	*** 0.000194

vs = versus
ns = no significant differences

Appendix Table S4.

Exact *P*-values for Figure EV3. Significant differences were determined using the unpaired Student's t test.

	Figure EV3 – Exact *P*-values
	MS + Naloxone vs MS + Naloxone + AZD
Body Wt Change	ns 0.4161
Body Tremors	** 0.0015
Jumping	* 0.0190
Mastication	*** P<0.0001
Paw Tremors	*** 0.0004
Piloerection	*** 0.0004
Ptosis	ns 0.0877
Wet Dog Shakes	** 0.0016

vs = versus
ns = no significant differences

Appendix Table S5.

Exact *P*-values for Figure EV4. Statistical differences were analyzed using ANOVAs followed by Duncan's *post hoc* comparisons.

	Figure EV4 – Exact *P*-values		
	WT vs HETERO Fyn$^{+/-}$	WT vs HOMO Fyn$^{-/-}$	HETERO Fyn$^{+/-}$ vs HOMO Fyn$^{-/-}$
**Body Wt Change**	ns 0.108359	** 0.002711	ns 0.060737
**Diarrhea**	ns 0.100100	*** 0.000328	# # 0.006680
**Grooming**	ns 0.644261	ns 0.806899	ns 0.806899
**Jumping**	ns 0.109699	* 0.017939	ns 0.298860
**Locomotor Activity**	ns 0.175171	* 0.042927	ns 0.385451
**Mastication**	ns 0.314930	* 0.013797	ns 0.080944
**Paw Tremors**	ns 0.366965	* 0.018425	ns 0.086846
**Wet Dog Shakes**	ns 0.058087	* 0.017195	ns 0.467082

vs = versus
ns = no significant differences

Appendix Table S6.

Exact *P*-values for Figure EV5. Statistical differences were analyzed using ANOVAs followed by Duncan's *post hoc* comparisons.

	Figure EV5 – Exact *P*-values
	Control vs Fyn shRNA
**Body Wt Change**	ns 0.918784
**Diarrhea**	ns 0.434601
**Grooming**	ns 0.949376
**Jumping**	** 0.009429
**Locomotor Activity**	ns 0.963819
**Mastication**	** 0.009917
**Paw Tremors**	* 0.011472
**Wet Dog Shakes**	ns 0.292145

vs = versus
ns = no significant differences

Appendix Supplementary Table 7.

Exact *P*-values for Appendix Figure S3. Statistical differences were analyzed using ANOVAs followed by Duncan's *post hoc* comparisons.

Appendix Figure S3 – Exact *P*-values	Saline + Naloxone vs MS + Naloxone	MS + Naloxone vs MS + Naloxone + AZD (2.5 µg)	MS + Naloxone vs MS + Naloxone + AZD (5 µg)	MS + Naloxone vs MS + Naloxone + AZD (10 µg)
**Body Wt Change**	# # 0.004501	ns 0.299552	ns 0.075019	** 0.006005
**Body Tremors**	# # # 0.000033	ns 0.635647	** 0.003917	** 0.005763
**Jumping**	# # 0.001151	** 0.008121	ns 0.09087	** 0.007544
**Rearing**	# # # 0.000035	** 0.004758	** 0.001170	*** 0.000074
**Mastication**	# # # 0.000034	ns 0.848985	*** 0.000081	*** 0.000056
**Piloerection**	# # # 0.000033	ns 0.294224	** 0.002126	** 0.006043
**Ptosis**	# # 0.001203	ns 0.096740	ns 0.130188	ns 0.460518

vs = versus
ns = no significant differences
