# Supplementary material for: Src‐dependent phosphorylation of μ‐opioid receptor at Tyr336 modulates opiate withdrawal
Source: EMBO Mol Med. 2017 Aug 18;9(11):1521–36. doi: 10.15252/emmm.201607324 (PMC5666313; doi:10.15252/emmm.201607324)
Supplement: Supplementary file 2 — Expanded View Figures PDF [file EMMM-9-1521-s002.pdf]

Expanded View Figures

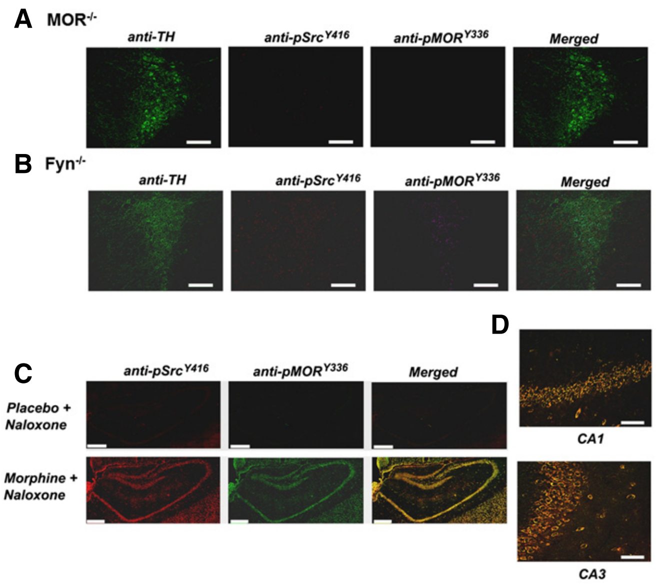

Figure EV1. Effect of naloxone-precipitated somatic opiate withdrawal on the increase in Src activation and MOR^{Y336} phosphorylation in WT, MOR$^{-/-}$, or Fyn$^{-/-}$ mice.

WT, MOR$^{-/-}$, or Fyn$^{-/-}$ mice were implanted with either placebo or morphine pellets and administered naloxone as detailed in the Materials and Methods.

A, B Minimal colocalization between pMOR^{Y336} and pSrc^{Y416} was observed in the TH^{+} neurons of the LC from MOR$^{-/-}$ (A) or Fyn$^{-/-}$ (B) mice treated with chronic morphine (pellets for 3 days) and injected with naloxone after morphine pellet removal on the 4th day. The green, red, and magenta colors represent TH, pSrc^{Y416}, and pMOR^{Y336}, respectively.

C Colocalization of pMOR^{Y336} (green) and pSrc^{Y416} (red) in the hippocampus of WT mice implanted with either placebo or morphine pellets. The yellow denotes overlap.

D A magnified view of the CA1 (upper) and CA3 (lower) regions from merged images of morphine- and naloxone-treated animals.

Data information: Scale bar = 70 μm for the LC and 100 μm for the hippocampus.

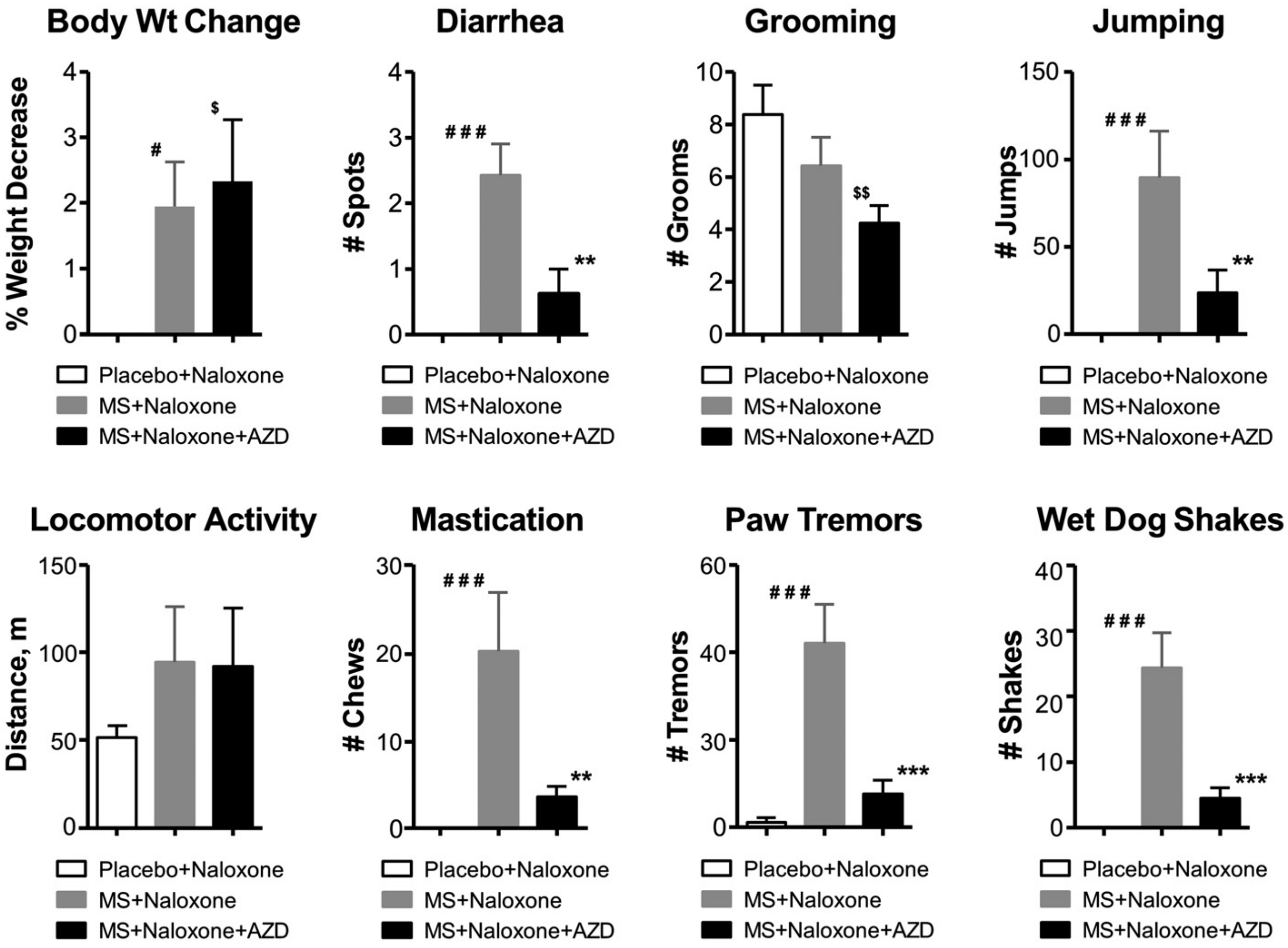

Figure EV2. Naloxone-precipitated withdrawal signs in WT mice (n = 8/group) i.c.v. injected with AZD0530.

This presentation allows a comparative view of all the withdrawal signs including jumping presented in Fig 5A. The inhibition of Src activity after the i.c.v. injection of 50 μg of AZD0530, induces a significant decrease in naloxone-precipitated diarrhea, jumping, mastication, paw tremors, and wet dog shakes (WDS). Body weight (Body Wt Change), grooming, and locomotor activity remain unchanged. Significant differences among the groups were determined using one-way ANOVA, followed by Duncans *post hoc* comparison. ***$P < 0.001$ and **$P < 0.01$ significant differences between the morphine-and-naloxone-treated mice (MS + Naloxone, $n = 8$) and morphine-naloxone-and-AZD-treated mice (MS + Naloxone + AZD, $n = 8$). $^{\#\#\#}P < 0.001$ and $^{\#}P < 0.05$ significant differences between the placebo-and-naloxone-treated mice (Placebo + Naloxone, $n = 8$) and morphine-and-naloxone-treated mice (MS + Naloxone). $^{\$\$}P < 0.01$ and $^{\$}P < 0.05$ significant differences between the placebo-and-naloxone-treated mice (Placebo + Naloxone) and morphine-naloxone-and-AZD0530-treated mice (MS + Naloxone + AZD0530). The bars and errors represent the means ± SEM. Exact P-values are in Appendix Table S3.

 © 2017 The Authors

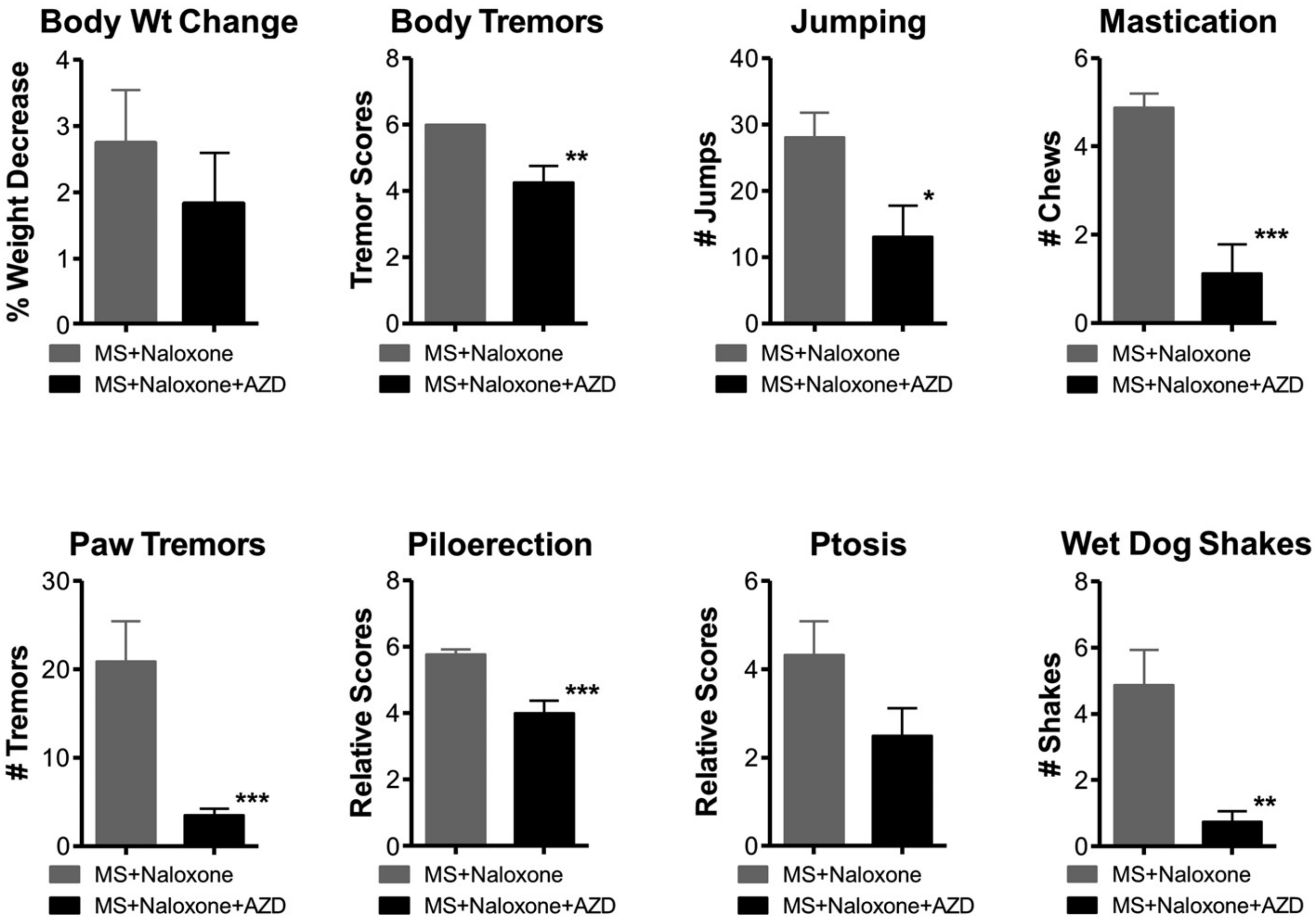

Figure EV3. Naloxone-precipitated withdrawal signs in WT mice in which AZD0530 was stereotaxically injected into the LC.

This presentation allows a comparative view of all the withdrawal signs including jumping presented in Fig 5B. The inhibition of Src activity following the injection of 7.5 μg/μl AZD0530 into the LC, induces a significant decrease in naloxone-precipitated body tremors, jumping, mastication, paw tremors, piloerection, and wet dog shakes. Body weight (Body Wt Change) and ptosis remain unchanged. Significant differences between the morphine-and-naloxone-treated mice (MS + Naloxone, $n = 11$) and morphine-naloxone-and-AZD-treated mice (MS + Naloxone + AZD, $n = 13$) were determined using the unpaired Students t-test. ***$P < 0.001$, **$P < 0.01$ and *$P < 0.05$ significant differences between MS + Naloxone and MS + Naloxone + AZD. The bars and errors represent the means ± SEM. Exact P-values are in Appendix Table S4.

© 2017 The Authors

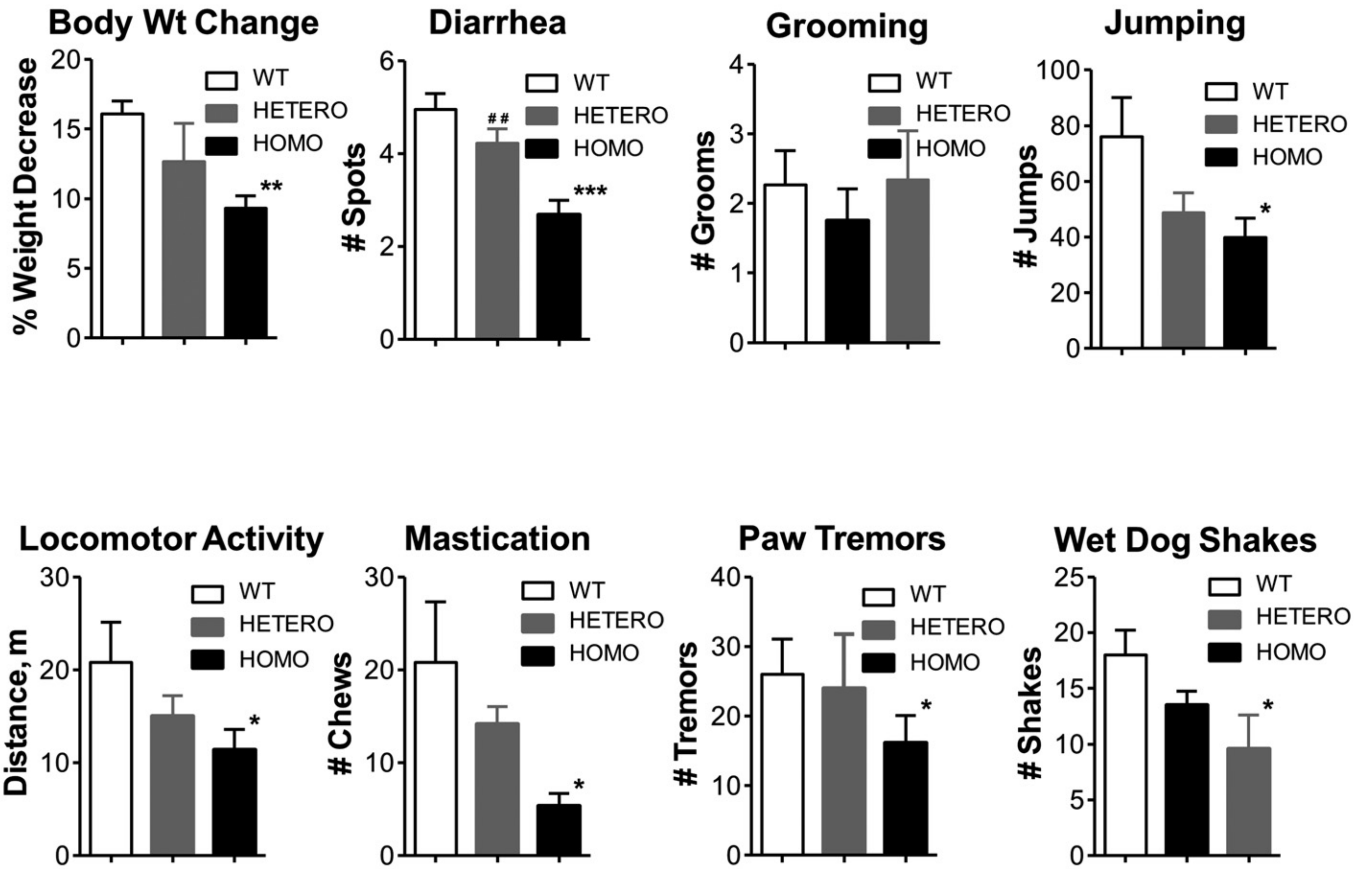

Figure EV4. Naloxone-precipitated withdrawal signs in WT, HETERO, and HOMO of Fyn$^{-/-}$ mice.

The significant reduction in body weight, diarrhea, jumping, mastication, paw tremors, and wet dog shakes observed in the HOMO Fyn$^{-/-}$ mice suggests the involvement of Fyn in the manifestation of these naloxone-precipitated withdrawal signs. Also, HETERO Fyn$^{+/-}$ mice display a significant attenuation of the number of jumps. Locomotor activity and grooming are not affected by the reduction or absence of Fyn in Fyn$^{+/-}$ or $^{-/-}$ mice, respectively. Significant differences among the groups were determined using one-way ANOVA, followed by Duncans *post hoc* comparison (n = 6/group). ***P < 0.001, **P < 0.01, and *P < 0.05 relative to WT mice; $^{\#\#}P$ < 0.01 significant differences between HETERO Fyn$^{+/-}$ mice and HOMO Fyn$^{-/-}$ mice. The bars and errors represent the means ± SEM. Exact P-values are in Appendix Table S5.

 © 2017 The Authors

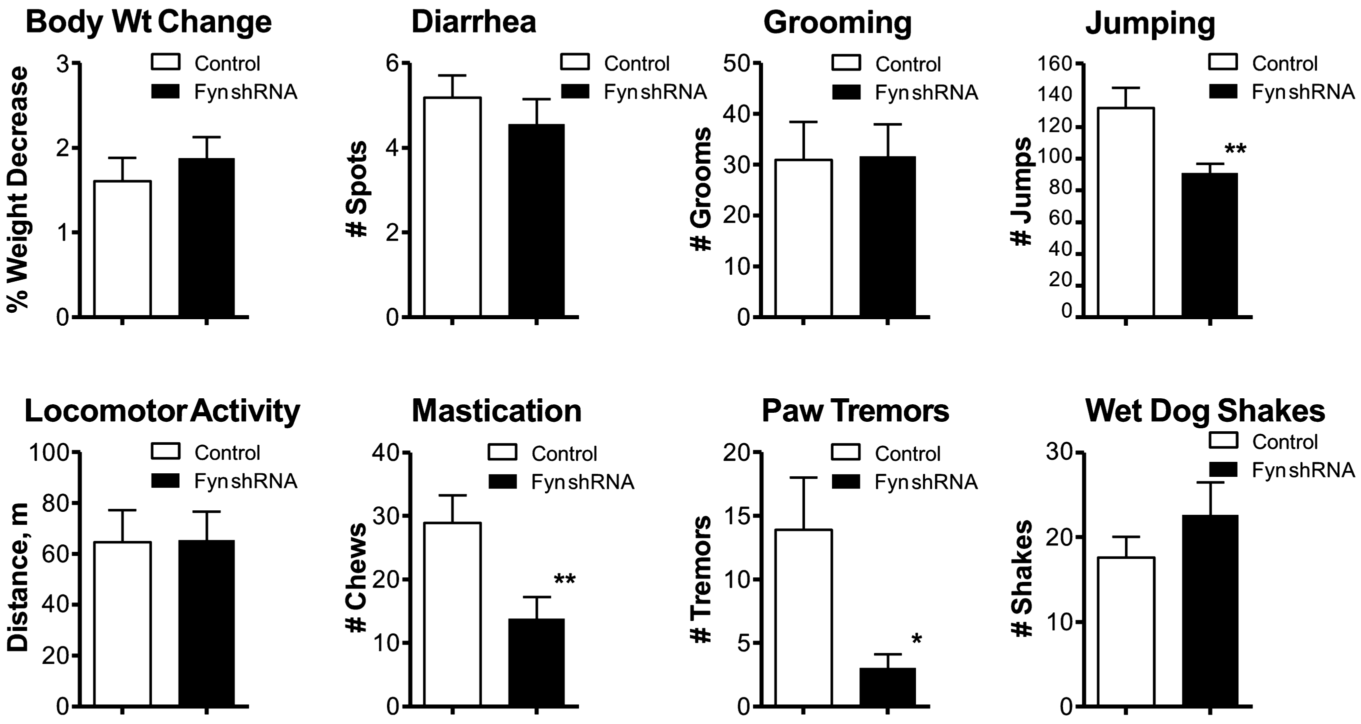

Figure EV5. Naloxone-precipitated withdrawal signs in WT mice injected in which control (GFP) or Fyn shRNA was injected into the LC.

One microliter of Fyn shRNA AAV2/9 virus (titer: 2.09×10^{12} v.g./ml) was injected bilaterally into the LC ($n = 12$) as described in the Materials and Methods. The same number of control mice was injected bilaterally with the control GFP virus. This presentation allows a comparative view of all the withdrawal signs including jumping presented in Fig 5E. Fyn knockdown induced a significant decrease in naloxone-precipitated jumping, mastication, and paw tremors. Body weight, diarrhea, grooming, locomotor activity, and wet dog shakes remain unchanged. Significant differences between the control (GFP-transferred) and Fyn shRNA-transferred WT mice were determined using Students *t*-test. $^{**}P < 0.01$ and $^{*}P < 0.05$ significant differences between the control (GFP-transferred) and Fyn shRNA-transferred WT mice. The bars and errors represent the means ± SEM. Exact *P*-values are in Appendix Table S6.

© 2017 The Authors
