# Supplementary figures and images for: Src‐dependent phosphorylation of μ‐opioid receptor at Tyr336 modulates opiate withdrawal
Source: EMBO Mol Med. 2017 Aug 18;9(11):1521–36. doi: 10.15252/emmm.201607324 (PMC5666313; doi:10.15252/emmm.201607324)

# Source data for Figure 1

1A

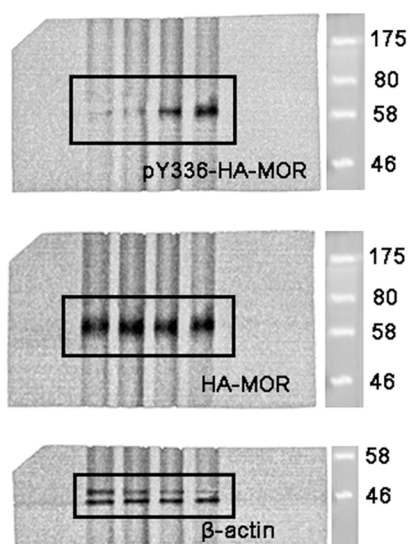

1B

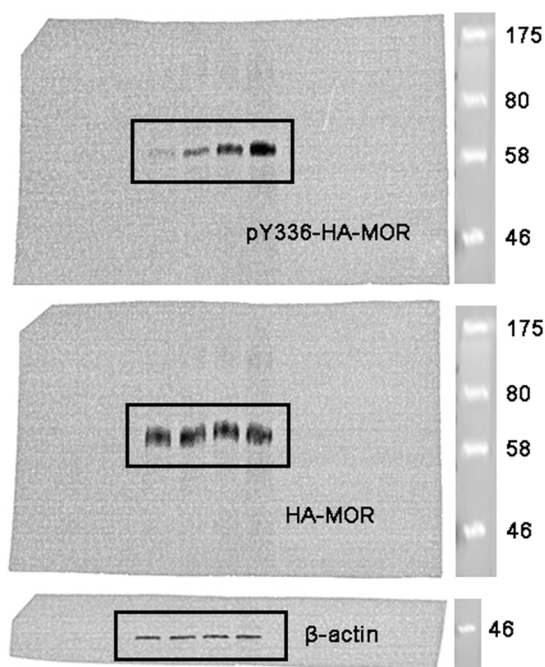

1C

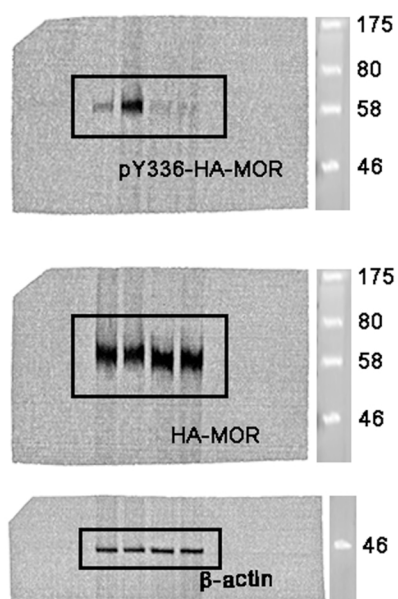

1D

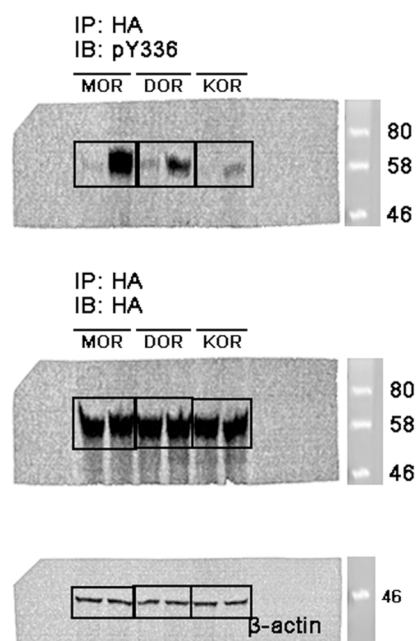

Supplement: Supplementary file 4 — Source Data for Figure 1 [file EMMM-9-1521-s003.pdf]

## Source data for Figure 3D

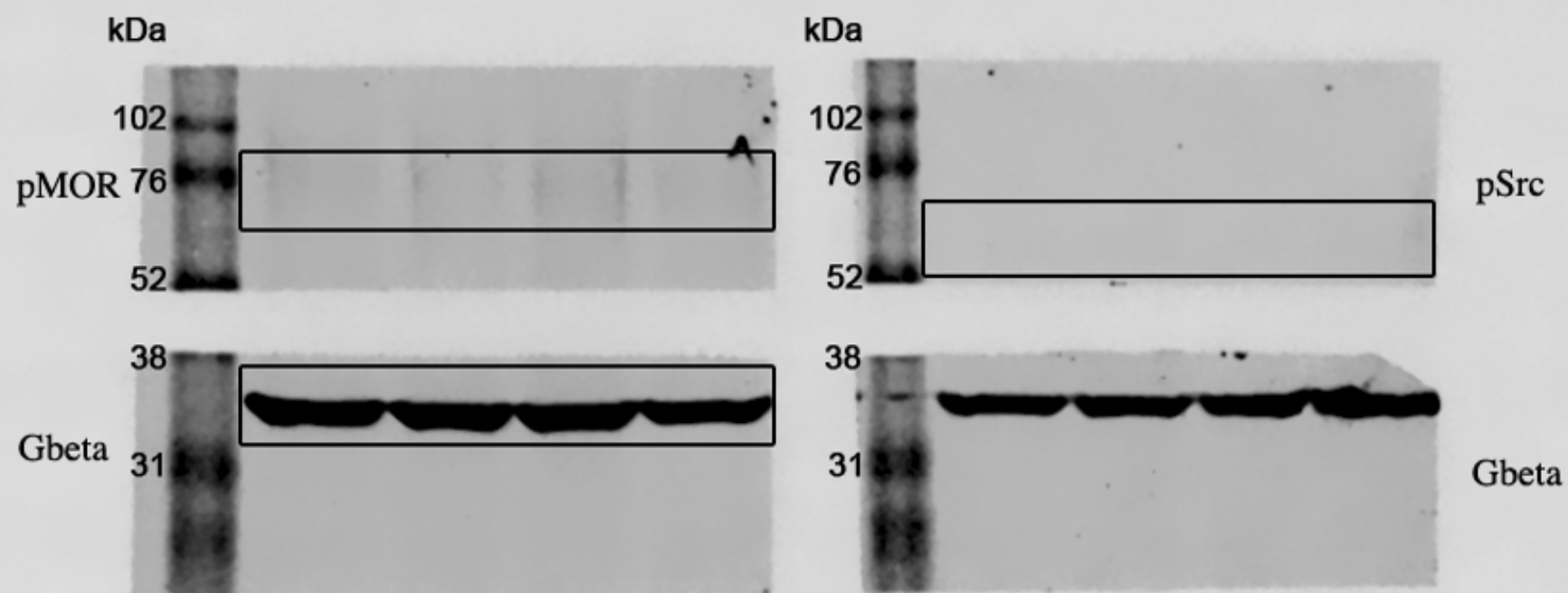

Supplement: Supplementary file 5 — Source Data for Figure 3 [file EMMM-9-1521-s004.pdf]
