# Supplementary material for: Src‐dependent phosphorylation of μ‐opioid receptor at Tyr336 modulates opiate withdrawal
Source: EMBO Mol Med. 2017 Aug 18;9(11):1521–36. doi: 10.15252/emmm.201607324 (PMC5666313; doi:10.15252/emmm.201607324)

Source data for Figure 4A

Naloxone-precipitated withdrawal WT mice

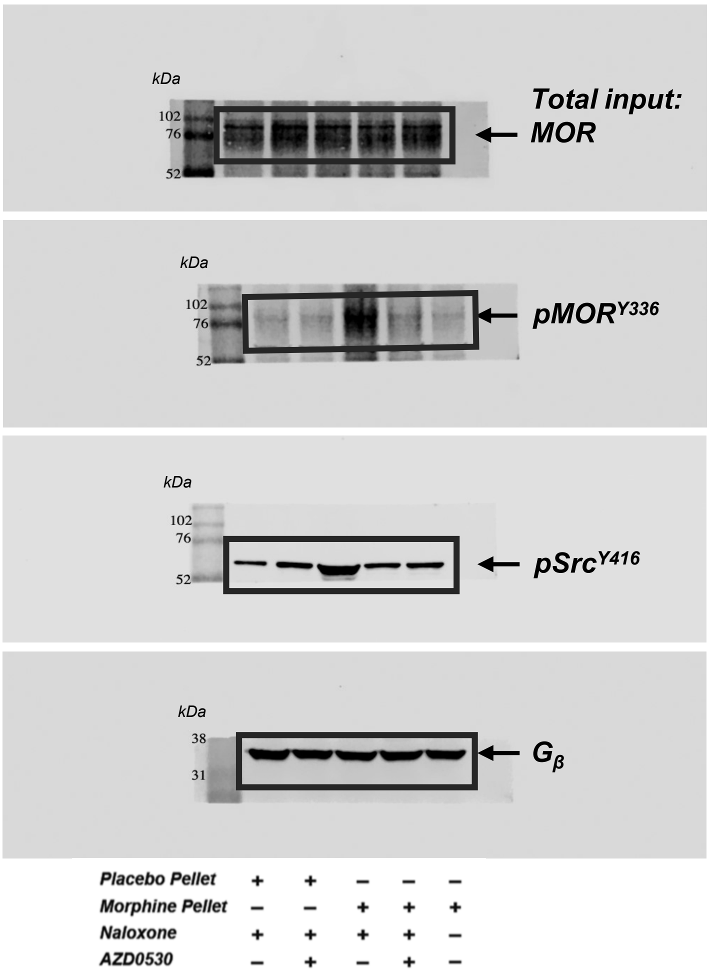

Pre-adsorption control

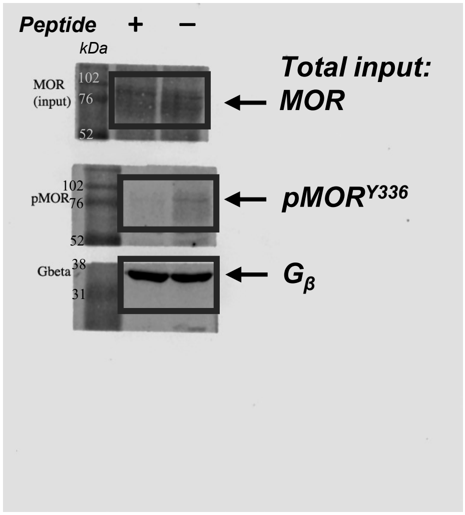

Fyn$^{-/-}$

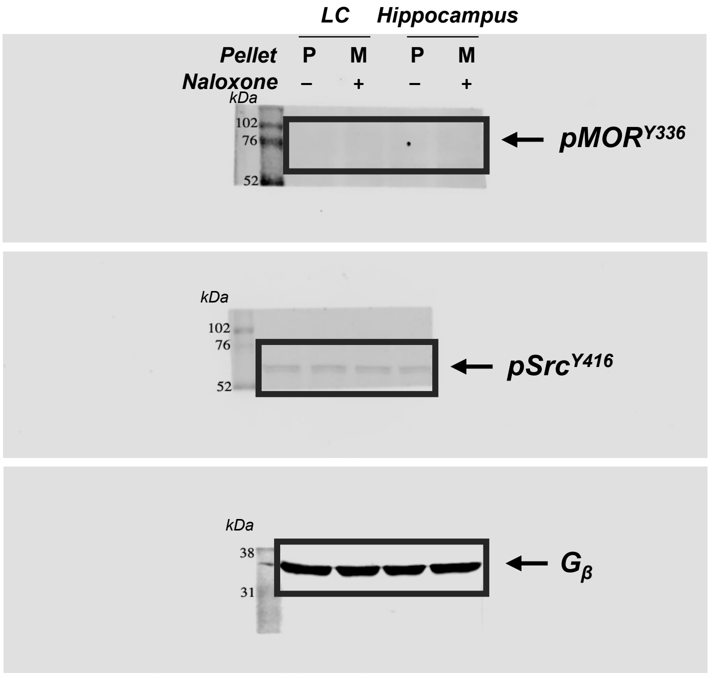

Supplement: Supplementary file 6 — Source Data for Figure 4 [file EMMM-9-1521-s005.pdf]
