# Supplementary material for: Src‐dependent phosphorylation of μ‐opioid receptor at Tyr336 modulates opiate withdrawal
Source: EMBO Mol Med. 2017 Aug 18;9(11):1521–36. doi: 10.15252/emmm.201607324 (PMC5666313; doi:10.15252/emmm.201607324)

Source data for Figure 7D

Original scan for Figure 7D

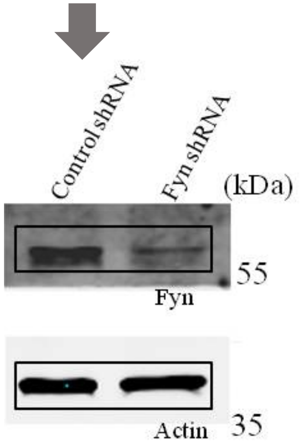

Same experiment: other uncropped and unprocessed scans with molecular weight markers.

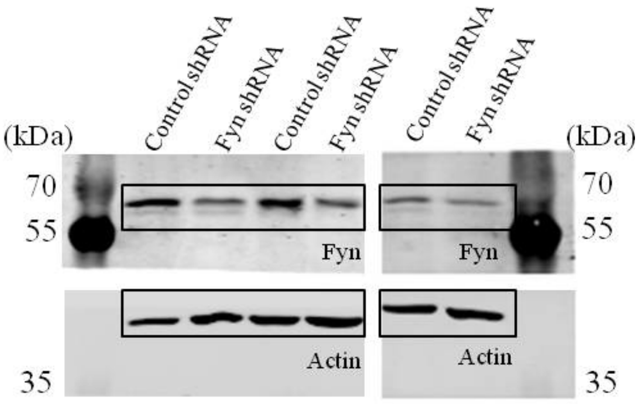

Same experiment: other uncropped and unprocessed scans with molecular weight markers.

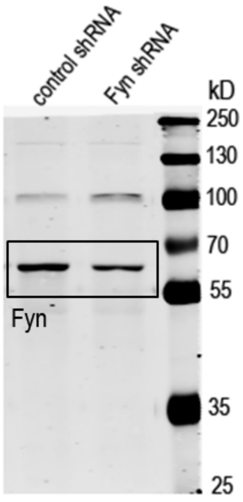

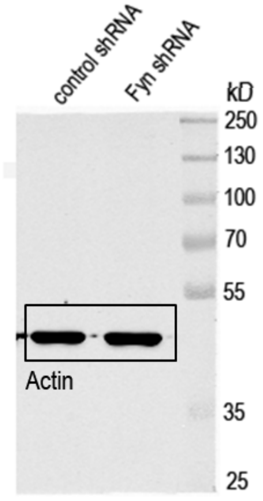

Supplement: Supplementary file 7 — Source Data for Figure 7 [file EMMM-9-1521-s006.pdf]
